# Molecular pathways that drive diabetic kidney disease

**DOI:** 10.1172/JCI165654

**Published:** 2023-02-15

**Authors:** Samer Mohandes, Tomohito Doke, Hailong Hu, Dhanunjay Mukhi, Poonam Dhillon, Katalin Susztak

**Affiliations:** 1 Renal, Electrolyte, and Hypertension Division, Department of Medicine;; 2 Institute for Diabetes, Obesity, and Metabolism;; 3 Department of Genetics; and; 4 Kidney Innovation Center; Perelman School of Medicine, University of Pennsylvania, Philadelphia, Pennsylvania, USA.

## Abstract

Kidney disease is a major driver of mortality among patients with diabetes and diabetic kidney disease (DKD) is responsible for close to half of all chronic kidney disease cases. DKD usually develops in a genetically susceptible individual as a result of poor metabolic (glycemic) control. Molecular and genetic studies indicate the key role of podocytes and endothelial cells in driving albuminuria and early kidney disease in diabetes. Proximal tubule changes show a strong association with the glomerular filtration rate. Hyperglycemia represents a key cellular stress in the kidney by altering cellular metabolism in endothelial cells and podocytes and by imposing an excess workload requiring energy and oxygen for proximal tubule cells. Changes in metabolism induce early adaptive cellular hypertrophy and reorganization of the actin cytoskeleton. Later, mitochondrial defects contribute to increased oxidative stress and activation of inflammatory pathways, causing progressive kidney function decline and fibrosis. Blockade of the renin-angiotensin system or the sodium-glucose cotransporter is associated with cellular protection and slowing kidney function decline. Newly identified molecular pathways could provide the basis for the development of much-needed novel therapeutics.

## Introduction

Diabetic kidney disease (DKD) refers to the clinical diagnosis of kidney disease attributed to diabetes based on the presence of albuminuria (>300 mg/d) and/or low estimated glomerular filtration rate (eGFR) (<60 cc/min) in patients with type 2 diabetes, but an even lower threshold of albuminuria can signify DKD in patients with type 1 diabetes. Diabetic nephropathy refers to the histological changes attributed to diabetes that are observed on kidney biopsies of patients with diabetes, including diffuse thickening of the glomerular basement membrane (GBM), mesangial expansion, and nodular sclerosis (Kimmelstiel-Wilson nodules) (1). Albeit these terms are often used interchangeably, here we will use the broader term of DKD to encompass the kidney disease attributable to diabetes.

Epidemiological studies indicate that around 40% of patients with diabetes develop DKD (2). However, this number varies depending on the DKD diagnostic criteria used (3). Overall, DKD remains the leading cause of end-stage kidney disease in the Western hemisphere, representing an enormous economic burden. Patients with DKD have increased cardiovascular disease risk and mortality. While the incidence of cardiovascular disease has markedly declined over the last three decades, the incidence of DKD has not declined similarly (4).

Our understanding of DKD remains incomplete, in part because animal models poorly recapitulate human DKD (5). The clinical disease manifestation appears to be heterogeneous; for instance, many patients progress in the absence of a large amount of albuminuria, and the histological characterization of these patients is still incomplete (6). DKD progression is variable, and no available biomarkers can accurately predict kidney function decline (7). In the last 3 to 5 years there have been some promising results in multiple clinical trials of drugs for treatment of DKD; while these drugs slow disease progression, they do not stop kidney function decline. Improved mechanistic understanding of DKD pathophysiology will be essential for new drug development. Below, we will review the current evidence implicating genetic, epigenetic, and metabolic alterations in DKD, linking these changes to microvascular, tubule, and podocyte dysfunction in disease progression.

## DKD has a strong heritable component

The genetic basis of DKD was first suggested by observation of the familial disease aggregation (8). Family-based early linkage studies identified suggestive genes and variants that were not replicated in later unbiased genome-wide association studies (GWAS). The Genetics of Nephropathy International Effort (GENIE) consortium (9), with a sample size of 6,000 subjects, identified two novel signals near the *AFF3* and the *RGMA*/*MCTP2* gene regions. The Surrogate Markers for Micro- and Macrovascular Hard Endpoints for Innovative Diabetes Tools (SUMMIT) Consortium (10), with a sample size of 12,000, only observed suggestive signals near *AFF3*, *CNTNAP2*, *NRG3*, *PTPN13*, and *GABRR1* genes (11). Increasing the sample size to 20,000 subjects with type 1 diabetes helped to identify a strong signal in the *COL4A3* gene that was associated with a thinner GBM and protection from albuminuria and DKD. Two other variants associated with cell adhesion were identified near the *COLEC11* and *DDR1* genes (12). The CKDGen, UKBB, and MVP consortia have performed extensive large eGFR GWAS studies with over 1 million subjects and identified more than 800 loci for eGFR (13). A subanalysis of subjects with diabetes (mostly type 2 diabetes) could not identify major differences in the genetic architecture of eGFR in subjects with or without diabetes (14).

In summary, the genetic architecture of albuminuria in DKD appears to be different from that of eGFR. The genetic architecture of eGFR is very similar in those with and without diabetes. The proteinuria phenotype seems to cluster with genes expressed by podocytes.

## Epigenetic changes are both heritable and metabolite-sensitive

Patients with diabetes who experience a period of poor glycemic control have higher kidney disease incidence even after several decades of good metabolic control (15), a phenomenon dubbed “metabolic memory.” Epigenomic changes are proposed to be responsible for the metabolic memory. Epigenome-modifying enzymes use substrates from the intermediate metabolism, rendering them sensitive to intracellular metabolite fluctuation (16). Indeed, culturing cells in high glucose causes changes in DNA cytosine methylation and histone modification (17). However, the epigenome is cell type specific, and in vitro cultured cells are different from in vivo, representing an important challenge for epigenetic studies.

### Methylation changes in DKD.

Methylation of DNA cytosines is an important epigenetic modification that can cause transcriptional repression. The importance of methylation in metabolic memory was suggested by the identification of methylation differences, for example in the *TXNIP* (thioredoxin-interacting protein) locus, in the Diabetes Complications Control Trial (DCCT) cohort (18). Mediation analysis indicated the causal role of *TXNIP* methylation and hyperglycemia in DKD progression (19). These methylation differences persisted in the follow-up EDIC cohort, suggesting their role in metabolic memory (20). In the Pima Indian cohort, methylation of 77 sites in blood samples was associated with GFR decline (21). In patients with diabetes among the Chronic Renal Insufficiency Cohort, an association between methylation changes and albuminuria, glycemic control, baseline eGFR, and eGFR decline was observed. The study also examined the relationship between genotype and methylation levels (methylation quantitative trait locus [meQTL]). Integration of GWAS and meQTL enabled identification of likely causal methylation changes for DKD development and novel risk genes (22).

Very few methylation studies analyzed changes in human kidney tissue samples. Among these, the earliest study by Ko et al. used an isoschizomer-based DNA digestion method and identified methylation changes in enhancer regions in diseased kidneys (23). Later, Gluck et al. identified loci showing differential methylation between healthy and diseased kidneys. Furthermore, kidney cytosine methylation changes improved the prediction of GFR decline, supporting their role in DKD (24). Higher methylation levels in enhancer regions correlated with lower expression of tubule-specific genes, suggesting a role of DNA methylation in epithelial dedifferentiation (25). The degree of methylation of the *TNF*α gene correlated with eGFR decline and fibrosis. Epigenetic editing of the *TNF* locus (using dCas9-Tet1) confirmed the causal role of the methylation of this region in TNF expression and disease development (5). Mouse models with diabetes displayed higher DNA methyltransferase 1 (DNMT1) levels. Genetic knockdown or pharmacological inhibition of DNMT1 lowered urinary albumin excretion and pathological features of DKD in mice (26). The role of other cytosine-methylating enzymes, such as Tet eleven hydroxylases (TET1 and TET2), was further supported by in vitro studies (27).

### Histone modifications in DKD.

Modifications of histone proteins such as acetylation, methylation, and sumoylation can alter the availability of the DNA for transcription. Histone modification analysis requires relatively large amount of tissue material, and only limited data are available for human kidney samples. Changes in the pattern of histone modification (histone 3 lysine 9, H3K9; lysine 4, H3K4) in DKD blood samples and their role in metabolic memory have been highlighted in the DCCT cohort (28) and in vitro studies (29). Histone modification enzymes include methyltransferases, demethylases, acetyltransferases, and histone deacetylases (HDACs). HDACs appear to play a role in fibrosis, inflammation, and podocyte and tubular injury, thereby contributing to DKD development (30, 31). In mice, a nonselective HDAC inhibitor (valproate) attenuated renal injury and fibrosis (32). Sirtuins are NAD^+^-dependent HDACs (33). Overexpression of Sirt1 or pharmacological stimulation (with resveratrol) protected from DKD (34, 35), and Sirt1 deficiency exacerbated renal injury in mouse models (36). Also in mouse models, Sirt6 has been shown to protect from podocyte injury via silencing of Notch signaling (37). Loss of enhancer of zeste homolog 2 (EZH2) decreased H3K27me3 levels in podocytes of models with diabetes, causing the activation of Notch signaling and podocyte dedifferentiation (38). Inhibition of the histone demethylase Jumonji 3 or UTX also attenuated podocyte injury in mice with diabetes (38).

### Chromatin accessibility alterations in DKD.

Single-nucleus RNA and open chromatin analysis of human DKD kidney samples was able to identify cell type–specific epigenetic changes by profiling chromatin accessibility. Reduced accessibility of glucocorticoid receptor binding sites and an injury-associated expression signature in the proximal tubule were identified. Chromatin accessibility might be regulated by genetic background and metabolic memory, which could preprogram proximal tubules to respond differently to external stimuli (39).

### Noncoding RNA expression in DKD.

Noncoding RNAs (ncRNAs) lack translational capability and account for approximately 95% of transcribed RNA, and they modulate gene expression. They are of particular interest as they can be modulated by novel RNA technologies. MicroRNA-21 (miR-21), miR-34-5p, miR-141, miR-370, miR-503, miR-184, miR-377, Let7, miR-25, miR-29, miR-93, miR-126, miR-130, miR-424, and miR-146 have been studied in more detail (40). MicroRNAs have been shown to regulate the inflammatory response, oxidative stress, metabolic abnormalities, immune response, and fibrosis through different signaling pathways and targets. Differential expression of long ncRNAs (lncRNAs) was also seen in DKD samples when compared with those with diabetes in the absence of kidney disease (41). Overexpression of TUG1 in podocytes was found to mediate mitochondrial function via PPARγ coactivator 1α (PGC-1α) (42, 43).

Genetic, biochemical, and clinical rationale supports the role of the epigenome in DKD development. Early surrogate cell-type data from in vitro and animal models indicate intricate changes in the epigenome in DKD. Large-scale characterization of cell type–specific changes will be essential to understand the role of epigenetic changes in DKD development, but studies are limited by the availability of human kidney samples.

## Changes in cellular metabolism in DKD

Despite the development of novel quantitative metabolomic tools, understanding metabolic changes in DKD remains a complex task. A small case-control study from the Joslin Diabetes Center identified changes in kidney filtration markers such as pseudouridine, essential amino acids, and derivatives in patients with DKD (44). Serum levels of seven modified metabolites (*C*-glycosyltryptophan, pseudouridine, *O*-sulfotyrosine, *N*-acetylthreonine, *N*-acetylserine, *N*_6_-carbamoylthreonyladenosine, and *N*_6_-acetyllysine) were associated with renal function decline in more than 100 patients with type 1 diabetes. Consistently, increased pseudouridine level was detected in the renal cortex of mice with DKD (45). Higher levels of leucine, valine, isoleucine, pseudouridine, and threonine and lower levels of citrate were found in the urine of 2,670 individuals with type 1 diabetes. 2-Hydroxyisobutyrate was associated with overall disease progression. Six amino acids and pyroglutamate were associated with progression from macroalbuminuria (46) (Table 1).

### Can lipid metabolites better predict DKD?

Large-scale metabolomic studies of the general population have established an inverse correlation between circulating acylcarnitines and eGFR (47). Acylcarnitines are intermediates of lipid metabolism that have been associated with insulin resistance. Blood samples from chronic kidney disease (CKD) patients showed a higher abundance of saturated C16–C20 free fatty acids and long-chain polyunsaturated complex lipids. Long-chain-to-intermediate-chain acylcarnitine ratio, a marker of β-oxidation efficiency, was lower with advancing CKD stages.

Complex changes have been observed in blood phospholipid species. Targeted serum lipidomic analyses in 669 individuals with type 1 diabetes showed that phosphatidylcholine and sphingomyelin species correlated with eGFR decline and albuminuria (48). A case-control study of 817 patients with type 1 diabetes showed that phosphatidylcholine was inversely and independently associated with rapid eGFR decline (49). Metabolomic analysis of patients with DKD identified increased urinary lysophosphatidylcholine with rapidly declining kidney function (50).

Genetic and animal model studies can help to propose causal relationships between metabolite levels and disease development. Genetic variants at the *NAT8* gene were strongly associated with CKD and also correlated with urinary levels of *N*-acetyltyrosine and *N*-acetylphenylalanine (51). NAT8 plays an important role in *N*-acetylation of metabolites. Kidney disease–associated *NAT8* variants influenced not only NAT8 levels but also serum acetyl amino acid levels (52). These studies suggest the potential role of NAT8 and acetylated amino acids in CKD development. Urinary 6-bromotryptophan levels were also associated with genetic variants and incident end-stage kidney disease (53). Similarly, serum 6-bromotryptophan level acts as a risk factor for CKD progression (54).

Similarly to patient samples, an early-stage DKD rat model also showed higher levels of lysophospholipids and sphingolipids, including ceramide and its derivatives (55). Oxidized phosphatidylcholine significantly correlated with creatinine levels in a rat kidney injury model (56). In summary, both human and animal model data indicate changes in phospholipids; however, their role in DKD is not clear.

Despite the major changes in metabolites in DKD, the causal role of most of these metabolites in DKD development remains unclear, with few exceptions, including phenyl sulfate (derived from gut microbiome), which is proposed to cause podocyte damage, leading to albuminuria in db/db mice (57). Future studies are warranted to define the role of metabolites in DKD development.

## DKD is a primary microvascular complication of diabetes

### Hemodynamic factors in early DKD: the role of hyperfiltration.

The kidney has an interesting double capillary system: arteries give rise to the glomerular capillary system, which then gives rise to the peritubular capillaries, and, finally, to venules. Glomerular pressure is maintained by the balance in tone between the afferent and efferent arterioles. Glomerular hyperfiltration is an early manifestation of DKD and most likely develops due to dysregulation of afferent and efferent arteriole tone (58). Glomerular hyperfiltration has been shown to be a risk factor for kidney function decline, cardiovascular disease, and mortality (59). The exact mechanism of hyperfiltration in diabetes is not well understood. Serum hyperglycemia will lead to higher glucose concentration in the ultrafiltrate. Glucose in the proximal tubules is reabsorbed via sodium-coupled mechanisms, which result in lower sodium chloride delivery to the distal nephron, such as the macula densa, a region of specialized cells that control the glomerular blood vessels. Low distal tubule sodium chloride delivery registers as low filtration resulting in a vasodilation of the afferent arteriole via the tubuloglomerular feedback mechanism (60). Angiotensin II can cause a relative efferent arteriole vasoconstriction, increasing the glomerular pressure and resulting in hyperfiltration (61). Glomerular hyperfiltration over time causes glomerulomegaly and increase the mechanical stretch of glomerular mesangial cells and podocytes (62). Podocyte hypertrophy follows glomerulomegaly to maintain slit diaphragm and to cover the expanded GBM (63).

### Changes in endothelial cells.

**Figure 1:**
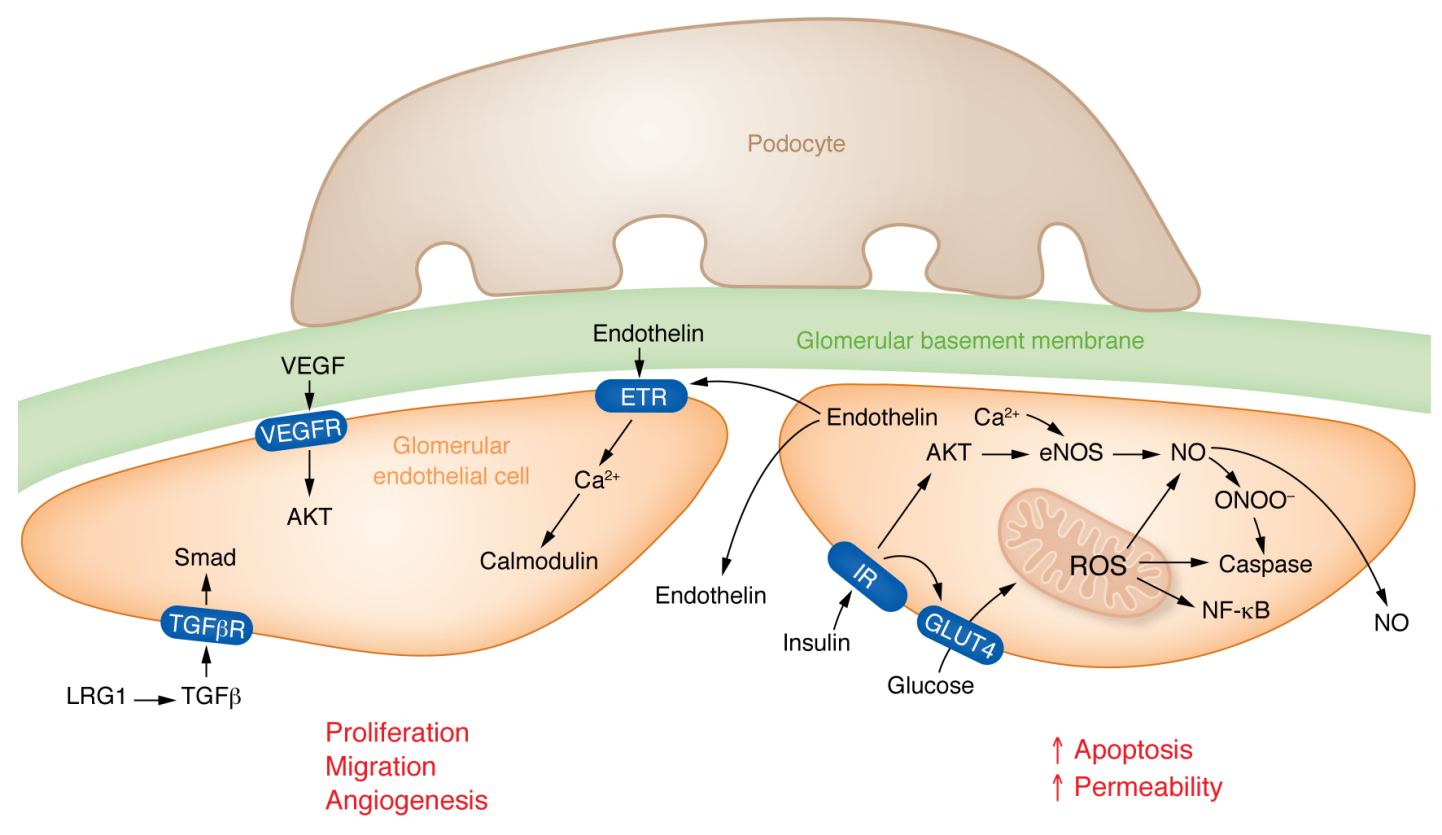
Changes of endothelial cells in diabetes. (Left) Early DKD is characterized by increased VEGFA expression, VEGFA released by podocytes binds to VEGFR receptors (VEGFR1/2) expressed on glomerular endothelial cells. In addition, VEGFA may also affect the balance between the production of the vasoconstrictive factor endothelin-1 (ET-1) and the vasodilatory factor nitric oxide (NO). By binding to its receptors VEGFR1 and VEGFR2 on GEnCs, VEGFA can stimulate NO production and inhibit ET-1 expression, thus exerting protective effects on the glomerulus. Glomerular endothelial cells release endothelin, which can signal to nearby endothelial cells via endothelin receptors (ETRs). LRG1 potentiates TGFβ signaling to activate Smad pathways in endothelial cells. All 3 signals shown in this cell induce proliferation, migration, and/or angiogenesis in endothelial cells in the DKD glomerulus. (Right) Elevated glucose levels and dysregulated insulin signaling promote increased mitochondrial reactive oxygen species (ROS) levels. Nitric oxide can react with endothelial ROS to release peroxynitrates (ONOO-). Increased oxidative stress will lead to apoptosis of glomerular endothelial cells and enhanced endothelial permeability — changes mostly observed in late DKD. IR, insulin receptor; TGFβR, TGFβ receptor; GLUT4, insulin sensitive glucose transporter 4.

DKD is a microvascular complication of diabetes; however, the exact pathways mediating endothelial dysfunction in DKD are poorly understood. Exposure of endothelial cells to high glucose can activate the polyol pathway, increase the production of reactive oxygen species (ROS) and mitochondrial dysfunction, and increase the expression of adhesion molecules promoting immune cell recruitment (64). In the kidney, glomerular endothelial cells (GEnCs) are specialized vascular cells that form the walls of the glomerular tufts and have important roles in renal homeostasis (Figure 1). GEnCs are covered by the glycocalyx, a network of endothelial polysaccharide layers. In animal models, the degree of albuminuria correlates with the loss of the glycocalyx (65). Heparanase-knockout mice (lacking an enzyme involved in degradation of the glycocalyx) showed protection from DKD (66); however, the role of the glycocalyx in patients with DKD remains controversial. Endothelial cell dysfunction can increase endothelial permeability and apoptosis and can cause loss of fenestration of GEnCs, leading to albuminuria. In streptozotocin-induced DKD mice, mitochondrial damage in GEnCs preceded podocyte damage, proteinuria, and glomerulosclerosis (67, 68). Mitochondria-targeted potent antioxidants prevented GEnC mitochondrial oxidative stress, loss of fenestrations, and loss of the endothelial glycocalyx (69).

### Angiogenic signals in the glomerulus.

Abnormal angiogenesis is a key feature of diabetic complications. Vascular endothelial growth factor A (VEGFA) released by podocytes binds to its receptors VEGFR1 and VEGFR2 expressed on GEnCs (70). VEGFA regulates the viability of GEnCs and it induces sprouting angiogenesis (71). In addition, VEGFA may also affect the balance between the production of the vasoconstrictive factor endothelin-1 (ET-1) and the vasodilatory factor nitric oxide (NO). Under physiological conditions, VEGFA induces NO production in GEnCs (72) and inhibits ET-1 expression, thus exhibiting a protective effect in the glomerulus (73, 74). VEGFA level might be higher in early DKD and reduced in late DKD, contributing to different pathologies. GEnCs are considered the principal source of endothelin-1 (ET-1) within the glomerulus. High levels of ET-1 inhibit NO production (75–77), and can cause a redistribution of the cytoskeleton (78, 79). NO also inhibits ET-1 expression and exerts protective effects (80, 81).

Recently, transcriptome profiling of GEnCs from mice with diabetes showed increased expression of leucine-rich α-2-glycoprotein 1 (LRG1) in DKD (82). LRG1 is a protein predominantly expressed by GEnCs that is involved in angiogenesis and the pathogenesis of DKD by enhancing endothelial transforming growth factor/activin receptor–like kinase 1 signaling. Global genetic ablation of LRG1 led to a reduction of oxidative damage, glomerular angiogenesis, and protection from DKD (83).

While mesangial expansion is an important component of DKD, changes in mesangial cells are poorly understood. Early studies using cultured mesangial cells highlighted the role of TGF-β, which induces mesangial matrix deposition (84). New spatially resolved single-cell studies will help to reexamine the interaction between the glomerular cells in diabetes.

## Podocyte dysfunction in DKD drives proteinuria

**Figure 2:**
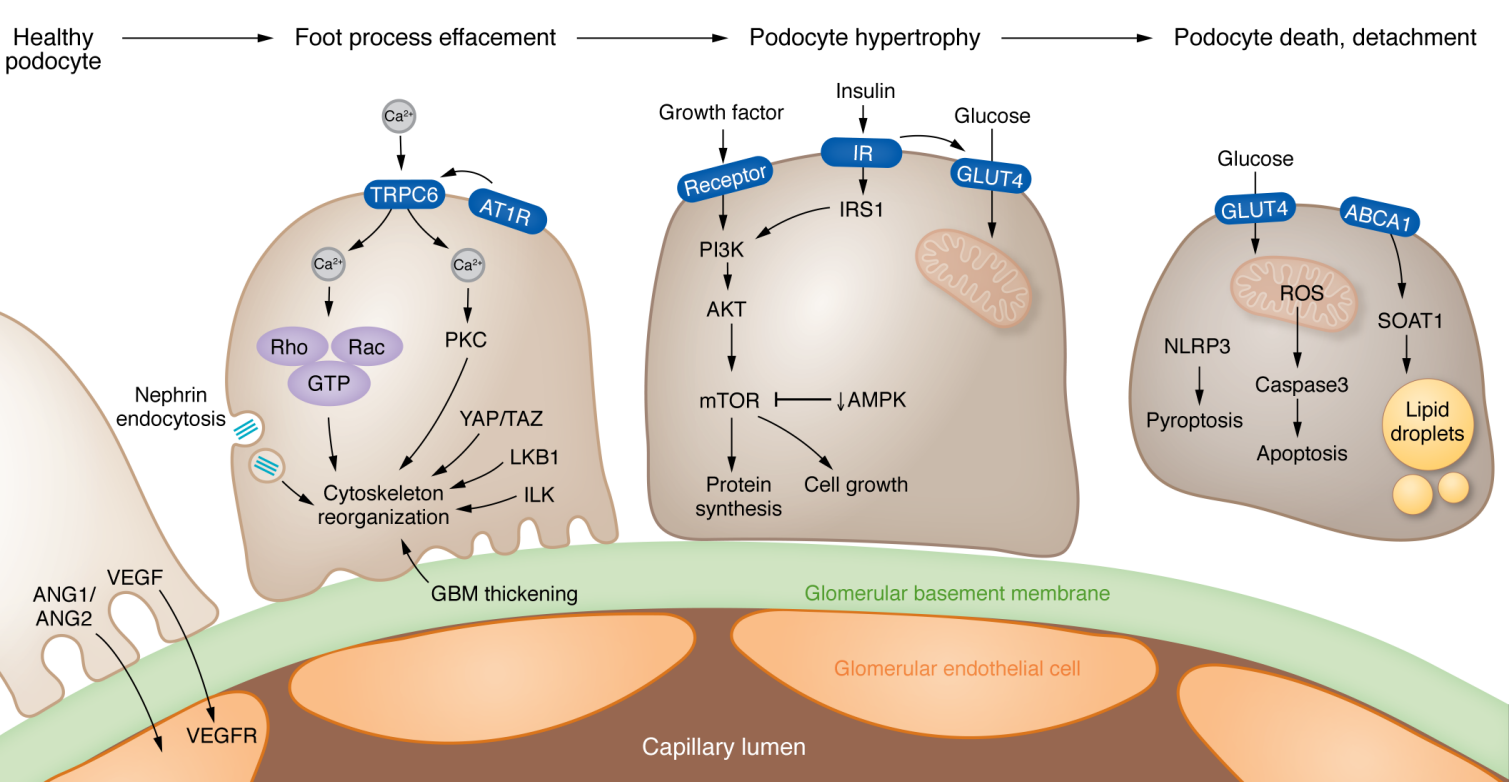
Mechanisms of podocyte dysfunction in DKD. (Left) Early stages of DKD lead to effacement of podocyte foot processes; multiple pathways contribute to the reorganization of the actin cytoskeleton, including TRPC6-mediated calcium influx activated by angiotensin (ANG) signaling, changes in nephrin endocytosis, and LKB1-associated signaling. In addition, mechanical stress pathways (YAP/TAZ), as well as thickening of the glomerular basement membrane (GBM), can induce integrin and downstream integrin linked kinase (ILK) activation. (Middle) Podocyte hypertrophy also characterizes the early stages of DKD. Growth factor and insulin signaling activates PI3K and mTOR pathways that regulate protein synthesis and cell growth. (Right) Later disease stages are characterized by podocyte death and dedifferentiation. The endoplasmic reticulum enzyme sterol-O-acetyltransferase-1 (SOAT1) facilitates the formation of cholesterol-enriched lipid droplets in podocytes. Dysregulated insulin signaling and high glucose levels trigger oxidative stress and apoptosis. NLRP3-mediated pyroptosis also contributes to podocyte loss. ABCA1, ATP-binding cassette A1; AT1R, angiotensin II receptor type 1; TRPC6, transient receptor potential channel 6.

Podocytes form the epithelial surface of the glomerulus, where filtration of molecules under 60 kDa takes place. Effacement of podocyte foot processes is linked to proteinuria and nephrotic syndrome development. Advanced DKD is usually associated with substantial, often nephrotic-range, proteinuria, highlighting podocytes’ key role in DKD (85) (Figure 2). Podocyte foot process effacement and enlargement are observed in early DKD, while podocyte loss is observed with more advanced disease (86). Podocytes are terminally differentiated cells unable to proliferate. More than 20% podocyte loss represents an irreversible step in DKD pathogenesis leading to glomerular scarring and development of end-stage kidney disease (87, 88).

### Mechanisms of podocyte dysfunction.

Some studies indicate that the high–growth factor milieu in prediabetic kidneys may lead to glomerular and podocyte enlargement resulting in albuminuria in rats (89, 90). mTOR integrates growth factor and insulin signaling to regulate cell growth, cell motility, cell survival, protein synthesis, autophagy, and transcription. mTORC1 signaling in podocytes appears to play a central role in the development of DKD (91). Recent studies have shown that complete ablation/inhibition of mTORC1 in podocytes resulted in increased vulnerability and glomerulosclerosis, in contrast to the reported therapeutic health benefits of mTOR inhibitors or just genetic lowering of mTOR level (92). mTOR-mediated podocyte hypertrophy is required to sustain glomerular integrity following podocyte loss, suggesting that the compensatory response can become maladaptive with time, leading to persistent damage. In addition to mTOR, both the serine-threonine kinase LKB1 and AMPK contribute to podocyte hypertrophy (93, 94).

Reorganization of the cytoarchitecture of podocytes — for example, changes in slit diaphragm proteins (nephrin) — serves as an important signaling platform. The TRPC channels TRPC5, TRPC6, and ORAI have been identified as key balancers of intracellular calcium levels in podocytes (95, 96). Cytosolic calcium levels regulate Rho and Rac proteins together with mechanical stretch–associated pathways (YAP/TAZ), leading to actin cytoskeleton reorganization in podocytes (97, 98). 

Changes in podocyte metabolism occur in early DKD. Podocytes appear to be sensitive to insulin and express the insulin-sensitive glucose transporter (GLUT4). In cultured podocytes, hyperglycemia will induce ROS production and subsequent activation of nuclear factor (erythroid-related 2)–like 2 (Nrf2), a redox-sensing transcription factor leading to apoptosis (99). Podocyte apoptosis correlated with the time of hyperglycemia in mouse models (99). Upregulation of enzymes in the glycolytic, sorbitol, methylglyoxal, and mitochondrial pathways has been shown to be protective in DKD. In particular, activation of pyruvate kinase 2 was shown to be protective against DKD by increasing glucose metabolic flux, reducing toxic glucose metabolites, and restoring mitochondrial function (100). Excessive lipid accumulation in podocytes, leading to lipotoxicity characterized by mitochondrial oxidative stress, inflammatory responses, actin cytoskeleton remodeling, insulin resistance, and endoplasmic reticulum stress has also been described in DKD (101). Cardiolipin accumulation is observed in patients carrying ATP-binding cassette A1 (ABCA1) loss-of-function mutations. ABCA1 deficiency is linked to cardiolipin-driven mitochondrial dysfunction in mice with podocyte-specific deletion of *Abca1* (102).

Changes in mitochondrial machinery further contribute to podocyte dysfunction, but podocyte-specific deletion of *Pgc1a*, *Drp1*, or *Tfam*, key molecular regulators of mitochondrial biogenesis, fission, and mitochondrial transcription, did not result in phenotypic changes in mice (103). On the other hand, transgenic expression of PGC-1α induced podocyte proliferation and rapid glomerulosclerosis (104). Hyperglycemia has been reported to alter podocyte metabolism by inducing DRP1-mediated mitochondrial fission through ROCK1, leading to detrimental effects in podocytes (105, 106).

Advanced stages of glomerulosclerosis are characterized by the reactivation of developmental genes, such as Notch and Wnt/β-catenin, driving the survival of the remaining podocytes (107–109). Transgenic mice with podocyte-specific stabilized β-catenin expression developed basement membrane thickening and mild albuminuria, resembling human DKD. As detailed above, metabolically driven epigenetic changes might drive the expression of Notch in podocytes, which in turn causes podocyte dedifferentiation.

In summary, functional and genetic studies highlighted the key role of podocytes in DKD, specifically in albuminuria. Podocyte hypertrophy might start before albuminuria, which seems to be adaptive early on but contributes to scarring later.

## Proximal tubule cell pathology correlates with GFR changes

**Figure 3:**
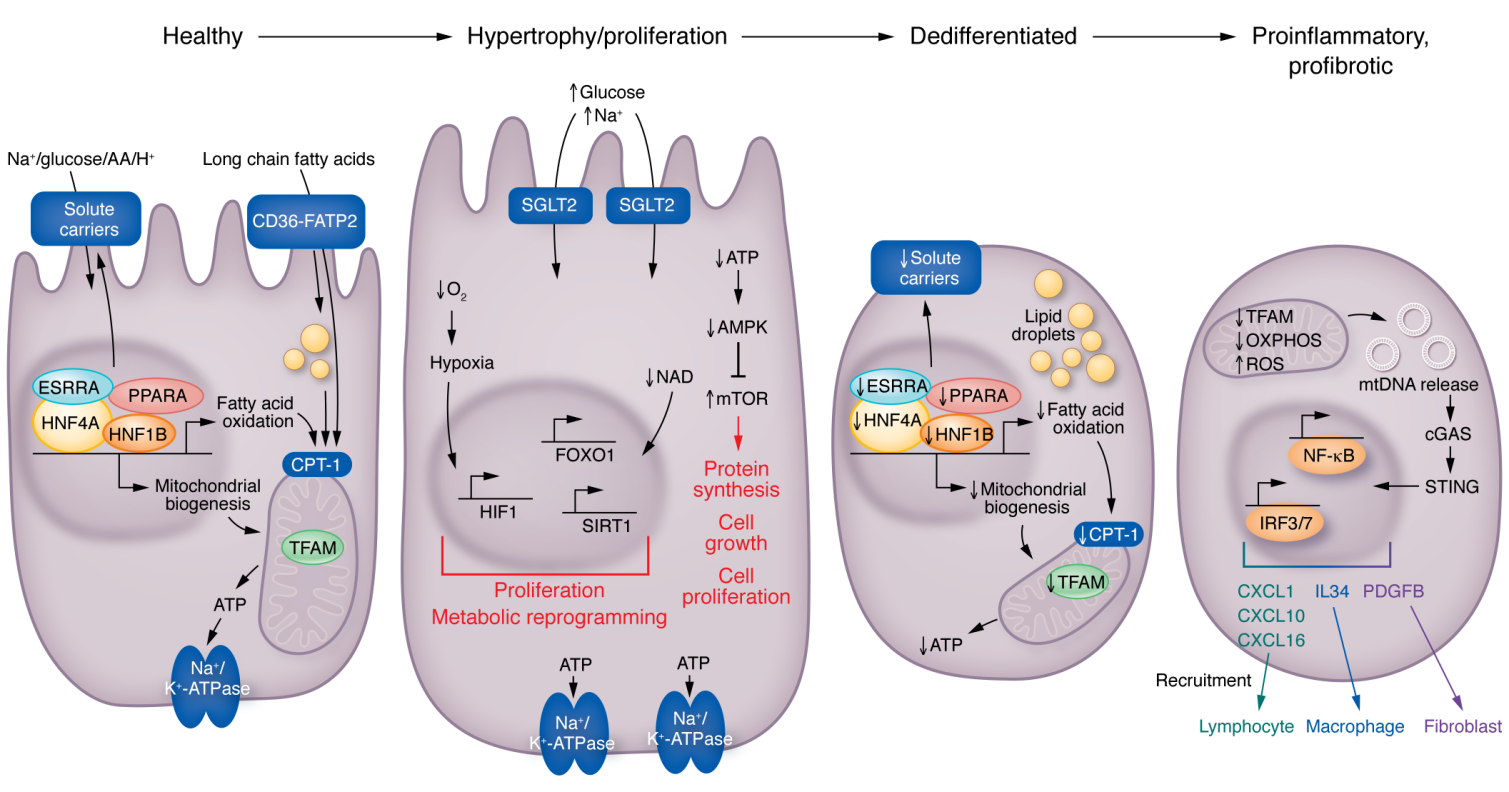
Changes in kidney proximal tubule cells in diabetes. Healthy PT cells utilize fatty acids and generate ATP via mitochondrial oxidative phosphorylation to support the Na^+^/K^+^ ATP-ase, which then creates a sodium gradient for sodium-mediated glucose, amino acid, or proton reabsorption. Increased tubule glucose presents an increased load for the basal Na^+^/K^+^ ATPase and the sodium-mediated glucose cotransporter; more oxygen and ATP are needed to meet this higher metabolic need. The higher AMP/ATP ratio is sensed by AMPK. This excess workload will result in reduced oxygen concentration (relative hypoxia), which is sensed by hypoxia-inducible factor (HIF). HIF activation will induce metabolic reprogramming, and, together with mTOR, will induce tubule cell proliferation and hypertrophy, proliferation, and kidney growth. Later stages of DKD are characterized by dedifferentiation of PT cells. Gene programs associated with mitochondrial biogenesis, fatty acid oxidation, mitochondrial function, and low ATP levels lead to the loss of solute carriers and cellular dedifferentiation. Finally, at later stages of DKD, severe mitochondrial damage will lead to the release of mitochondrial DNA (mtDNA) into the cytosol. Cystosolic mtDNA is then sensed by nucleotide sensing pathways, such as cGAS and stimulator of interferon genes (STING), inducing the activation of IRF3/7 and NF-κB pathways and resulting in transcription of cytokines, growth factors, and downstream immune cell recruitment and fibroblast activation. CPT-1, carnitine palmitoyltransferase 1A; ESRRA, estrogen-related receptor alpha; FATP2, fatty acid transport protein 2; HNF1B, hepatocyte nuclear factor 1B; IRF3/7, inferferon regulatory factor 3/7.

Recent genetic studies paradoxically indicated the role of proximal tubule (PT) cells, not glomerular cells, in determining glomerular filtration. Mapping of more than 800 loci associated with kidney function prioritized the role of hundreds of genes (13). Most identified eGFR-associated genes are expressed by kidney PT cells (110). The potential role of kidney PTs has already been observed in animal models and human studies (111). Hyperfiltration and kidney hypertrophy are the earliest changes of DKD. PT cells are mostly responsible for this renal enlargement and hyperfiltration (112). In advanced DKD, loss of kidney PT cells correlates with eGFR decline (Figure 3).

### Defective mitochondrial metabolism in kidney disease.

PT cells reabsorb 5 mM glucose from the 180 liters of primary filtrate that the kidneys produce each day. Most likely, to protect against glucose-induced metabolic stress (113), PT cells exclusively use fatty acids as their energy source (114). The molecular mechanism of hyperglycemia-induced tubule growth in early diabetes is not fully understood. Increased glucose reabsorption by PT requires a large amount of ATP and oxygen, representing a considerable workload for PT cells (115, 116). This increased workload lowers cellular ATP, NADH, and NADPH content and reduces intracellular oxygen concentration — creating relative cellular hypoxia. Hypoxia is sensed by the hypoxia-sensing pathway hypoxia-inducible factor (HIF), and the higher AMP/ATP by the AMP-activated protein kinase (AMPK) and mTOR activation, causing PT hypertrophy, proliferation, and kidney growth (ref. 117; Figure 3).

PT cells have one of the highest mitochondrial densities and produce energy by fatty acid oxidation (FAO) and oxidative phosphorylation (OXPHOS). Reduced and inefficient FAO is thought to be a major mechanism underlying tubular injury and fibrosis. A landmark study (118) demonstrated that FAO transcripts and their upstream transcriptional regulators such as PPARA, PGC-1α, LXR, FXR, and ESRRA were lower in CKD and DKD subjects with fibrosis. Pharmacological or genetic increase of PGC-1α (118, 119) or PPARA ameliorated CKD, while genetic deletion of *Ppara* (120), *Esrra* (121), and *Pgc1a* (122) was associated with more severe disease. A recent study showed that renal tubule–specific overexpression of the outer mitochondrial membrane protein carnitine palmitoyltransferase 1A (CPT1a) reduced fibrosis and renal pathology in mice (123). CPT1a overexpression improved mitochondrial morphology and increased FAO, supporting the hypothesis that FAO defect is a key driver of kidney fibrosis. In addition to the FAO defect, increased lipid accumulation by the fatty acid transporters CD36 (118) and FATP2 contributes to lipid accumulation and tubule lipotoxicity (124). Genetic deletion or pharmacological inhibition of FATP2 protected mice from tubulointerstitial fibrosis and DKD, indicating the role of lipid uptake in PT cells (125, 126).

Bulk gene expression analysis has indicated the dysregulation of a few common pathways in DKD, such as changes in FAO and OXPHOS, and also the unexpected activation of immune cells, genes, and pathways (118). It appears that some of proximal tubule cells take on an injured, profibrotic, or proinflammatory phenotype in disease states, expressing and releasing a variety of cytokines and chemokines (111, 127–129). Animal model experiments indicate impressive disease protection following genetic or pharmacological blockade of these immune pathways, but the effectiveness of cytokine inhibition in patients with DKD remains to be established (130).

The relationship between the metabolic disturbances and the injured proinflammatory, profibrotic PT cell phenotype development in disease states is not fully understood. Mitochondrial changes might provide new clues to explain this relationship. Mitophagy plays an important role in clearing damaged mitochondria (131). Defects in mitophagy can cause excess accumulation of ROS from damaged mitochondria (132). Increased cellular ROS can induce inflammatory cell death pathways such as ferroptosis (133). Inhibitors of ferroptosis have shown benefits in mouse models (134, 135). Peroxidation of lipids by ROS represents the root cause of ferroptosis. The loss of antioxidant genes such as *GPX4* and activation of *ACSL4* have been observed in mouse models and patients with DKD (135). Pyroptosis is another inflammatory cell death mechanism that is strongly dependent on NLRP3, gasdermin, and caspase-1 activation, leading to cleavage of IL-1β and cellular death (136, 137). Inflammasome-mediated cell death has been shown to contribute to DKD by several groups (138, 139). More severe damage can lead to the release of mitochondrial DNA (mtDNA) into the cytosol. The release of mtDNA (140, 141) into the cytosol in kidney tubule cells causes the activation of the cytosolic cGAS/stimulator of interferon genes (STING) DNA sensing pathway and activation of IRF3/7 and NF-κB pathways, resulting in transcription of cytokines and subsequent immune cell recruitment. Ablation of STING ameliorated kidney fibrosis in mouse models of CKD (142).

Single-cell expression analysis further highlighted PT cell plasticity. PT cells show the greatest differences in gene expression in disease states (142, 143). Interestingly, several groups have identified a unique subpopulation of PT cells that are mostly detectable in disease states (39, 121). These injured PT cells express VCAM1 or KIM1 and secrete a variety of cytokines, such as IL-34, responsible for macrophage influx; a variety of chemokines responsible for lymphocyte influx; and PDGFB and IHH, responsible for myofibroblast activation (144). Recently it has been shows that injured PT cells release CXCL1, attracting basophils. Basophils are the main source of IL-6 and IL-33, attracting Th17 cells and contributing to fibrosis development (145). Indeed, tubule epithelial cell dedifferentiation and the influx of immune cells and myofibroblast activation are common features of fibrosis-associated kidney function decline in DKD, as in other forms of progressive CKD (146). Recent spatial transcriptomic analysis of human DKD samples highlighted two types of injured PT cells, one with changes in metabolism and another with a proinflammatory phenotype (144). These cells were close to fibrotic areas. Indeed, the fibrotic area contained a very diverse cell population including myeloid and lymphoid cells, endothelial cells, and a variety of stromal cells and fibroblasts. These cells have complex cell-cell interactions and likely play a key role in fibrosis development. Consistent with these observations, single-cell studies on fibrotic human kidney samples indicated that fibroblast activation plays a key role in matrix accumulation (147). Overall, the fibrotic stroma plays an important role in progression, as the gene signature derived from this stroma was able to predict kidney function decline in a large cohort of samples (148).

In summary, PT cells play a key role in DKD development. PT cell hypertrophy and proliferation are observed at early stages, which is likely needed to match the increased metabolic demand and hyperfiltration. Changes in metabolism and mitochondrial integrity drive progressive PT loss, dedifferentiation, and tubulointerstitial inflammation development.

## Current and future mechanism-based therapies

The ACCORD and VADT studies demonstrated that controlling glycemia alone is not sufficient to eliminate diabetic complications and improve survival (149). This has resulted in a major shift in diabetes therapeutics registration, and at present, medications should focus on complication prevention, not just glucose lowering. Kidney disease is a key driver of mortality in patients with diabetes. More than 20 years ago, blockers of the renin-angiotensin-aldosterone system (RAAS) became a mainstay therapy, as they were shown to lower composite renal outcomes (death, dialysis, doubling of serum creatinine) by 20% (150). RAAS inhibitors lower blood pressure, which is likely important for their therapeutic action. They also acutely lower eGFR, which has mostly been attributed to a reduction in efferent glomerular artery tone and hyperfiltration (151). Recent GWAS studies identified genetic variants that modulate the expression of angiotensin-converting enzyme and the angiotensinogen genes in human kidney PT cells (152).

Inhibitors of SGLT2 (SGLT2i) have recently gained attention, as they lower composite kidney outcomes by 40% in addition to lowering heart failure mortality and death (153–155). While these drugs selectively target SGLT2, which is exclusively expressed by PT cells, the full extent of their mechanism of action remains to be elucidated. SGLT2i blocks glucose absorption in PT cells, thereby delivering more glucose, sodium, and chloride to the macula densa (112, 156). Increased chloride delivery can lower efferent artery pressure and acutely lower GFR and hyperfiltration by a tubulo-glomerular feedback mechanism. In the long term, lowering the energy required for glucose reabsorption lowers the energy need of PT cells and improves their function. Some studies suggest that SGLT2is are associated with mild ketosis, and ketone bodies might improve heart and PT function, as they are preferentially metabolized (157, 158). PPARA agonists similarly improve FAO in animal models, and while their effect on eGFR in the ACCORD study was inconclusive, they lowered albuminuria and were associated with slower GFR decline (159). Single-cell analysis of a murine DKD model showed that combination therapy such as SGLT2i and angiotensin-converting enzyme inhibitor had a non-overlapping synergistic effect reducing PT cell injury, suggesting that combination therapies may need to be adopted (160). With its recent approval, the nonsteroidal mineralocorticoid receptor antagonist finerenone became another new drug in our pharmacological armamentarium (161). Finerenone antagonizes the mineralocorticoid receptor in the distal nephron and protects tubule cells from taking on a profibrotic phenotype (162). The addition of finerenone also led to a reduction of albuminuria in the ARTS-DN study (163) and decreased progression of kidney disease and improved cardiovascular outcomes in the FIDELIO-DKD and FIGARO-DKD studies (161, 164). The use of finerenone has been recommended for its renal and cardiovascular benefits in patients (165). Hyperkalemia might be less frequent with finerenone compared with spironolactone, but is one side effect (166). GLP1 receptor agonists are the recommended second-line drugs for lowering blood glucose in DKD because of their demonstrated improvement of cardiovascular outcomes (165, 167). GLP1 receptor agonists lower albuminuria, but their effect on composite renal outcome is not yet available (168). Endothelin receptor blockers showed benefit in clinical trials using selected patients; however, they are not currently approved for DKD (169). No drugs have been approved that would target the podocytes.

In summary, recent positive clinical trial results are encouraging. The results are consistent with genetic and molecular studies. The present drugs mostly target the kidney PT cells and are associated with GFR protection. Future studies will analyze whether targeting other cell types such as podocytes, immune cells, or fibroblasts is associated with therapeutic benefits.

## Conclusion

DKD remains the leading cause of CKD and end-stage kidney disease and the major driver of mortality in patients with diabetes. DKD is defined by GBM thickening and, clinically, albuminuria and low GFR. Both genetic and recent epidemiological studies indicate differences in disease-driving mechanisms of albuminuria and GFR. Biochemical and epidemiological data suggest the role of epigenetic mechanisms; however, future large-scale studies will be needed to fully characterize the DKD metabolome and epigenome. Hyperglycemia drives metabolic changes in endothelial cells and podocytes, contributing to complex changes in cell-cell interaction and angiogenic pathways. Podocyte hypertrophy and changes in actin reorganization are critical adaptive mechanisms in glomerulomegaly but also a major contributor to damage over years and decades. Genetic, molecular, and pharmacological studies highlighted the key contribution of PT cells to eGFR decline and DKD development. PT metabolism and mitochondrial changes induce the development of a profibrotic proinflammatory PT cells, which appears to be critical for disease progression. Newly introduced drugs target PT cells and are shown to have a major impact on DKD development. Despite our remarkable therapeutic success over the last couple of years, a large number of patients continue to progress. Understanding disease-driving mechanisms provides new opportunities for precision therapeutics to treat this devastating disease condition.

## Figures and Tables

**Table 1:**
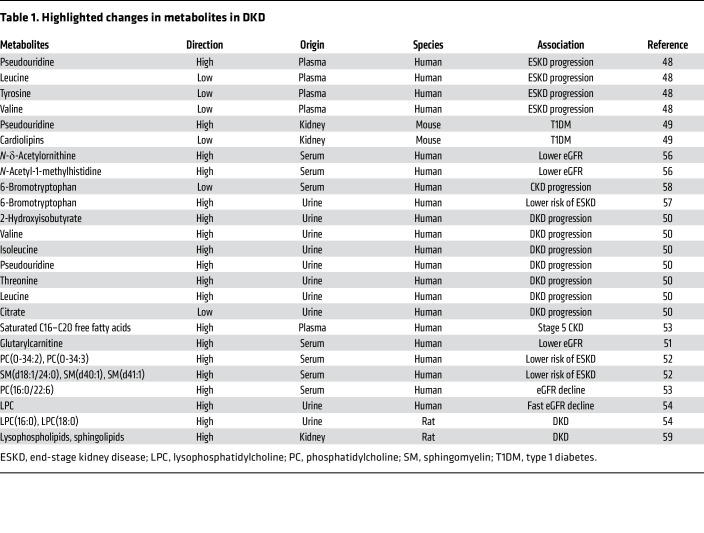
Highlighted changes in metabolites in DKD

## Version 1 02/15/2023

Electronic publication
